# Loss of AP-5 results in accumulation of aberrant endolysosomes: defining a new type of lysosomal storage disease

**DOI:** 10.1093/hmg/ddv220

**Published:** 2015-06-17

**Authors:** Jennifer Hirst, James R. Edgar, Typhaine Esteves, Frédéric Darios, Marianna Madeo, Jaerak Chang, Ricardo H. Roda, Alexandra Dürr, Mathieu Anheim, Cinzia Gellera, Jun Li, Stephan Züchner, Caterina Mariotti, Giovanni Stevanin, Craig Blackstone, Michael C. Kruer, Margaret S. Robinson

**Affiliations:** 1 Cambridge Institute for Medical Research, University of Cambridge, Cambridge, UK,; 2 Inserm U 1127, CNRS UMR 7225, Sorbonne Universités, UPMC Univ Paris 06 UMR S_1127, Institut du Cerveau et de la Moelle épinière, Paris F-75013, France,; 3 Ecole Pratique des Hautes Etudes, Paris F-75014, France,; 4 Sanford Children's Health Research Center, Barrow Neurological Institute and Ronald A. Matricaria Institute of Molecular Medicine, Phoenix Children's Hospital, Sioux Falls, SD, USA,; 5 Cell Biology Section, Neurogenetics Branch, National Institute of Neurological Disorders and Stroke, National Institutes of Health, Bethesda, MD, USA,; 6 APHP, Department of Genetics, Pitié-Salpêtrière Hospital, Paris F-75013, France,; 7 Département de Neurologie, Hôpital de Hautepierre, Strasbourg, France,; 8 Genetics of Neurodegenerative and Metabolic Diseases Unit, IRCCS-Fondazione Istituto Neurologico Carlo Besta, Milan 20133, Italy,; 9 Department of Neurology, Vanderbilt Brain Institute and Centre for Human Genetics Research, Vanderbilt University School of Medicine, 1161 21th Avenue South, Nashville, TN, USA,; 10 Department of Human Genetics and Hussman Institute for Human Genomics, Miller School of Medicine, University of Miami, Miami, FL, USA,; 11 Barrow Neurological Institute & Ronald A. Matricaria Institute for Molecular Medicine, Phoenix Children's Hospital, Phoenix, AZ and; 12 Department of Child Health, University of Arizona College of Medicine, Phoenix, AZ

## Abstract

Adaptor proteins (AP 1–5) are heterotetrameric complexes that facilitate specialized cargo sorting in vesicular-mediated trafficking. Mutations in *AP5Z1*, encoding a subunit of the AP-5 complex, have been reported to cause hereditary spastic paraplegia (HSP), although their impact at the cellular level has not been assessed. Here we characterize three independent fibroblast lines derived from skin biopsies of patients harbouring nonsense mutations in *AP5Z1* and presenting with spastic paraplegia accompanied by neuropathy, parkinsonism and/or cognitive impairment. In all three patient-derived lines, we show that there is complete loss of AP-5 ζ protein and a reduction in the associated AP-5 µ5 protein. Using ultrastructural analysis, we show that these patient-derived lines consistently exhibit abundant multilamellar structures that are positive for markers of endolysosomes and are filled with aberrant storage material organized as exaggerated multilamellar whorls, striated belts and ‘fingerprint bodies’. This phenotype can be replicated in a HeLa cell culture model by siRNA knockdown of AP-5 ζ. The cellular phenotype bears striking resemblance to features described in a number of lysosomal storage diseases (LSDs). Collectively, these findings reveal an emerging picture of the role of AP-5 in endosomal and lysosomal homeostasis, illuminates a potential pathomechanism that is relevant to the role of AP-5 in neurons and expands the understanding of recessive HSPs. Moreover, the resulting accumulation of storage material in endolysosomes leads us to propose that AP-5 deficiency represents a new type of LSDs.

## Introduction

Adaptor proteins (AP 1–5) are ubiquitously expressed protein complexes that facilitate vesicle-mediated intracellular sorting and trafficking of selected transmembrane cargo proteins (1). To date, mutations in components of all five AP complexes have been reported to impact human health [for which the term ‘adaptinopathies’ has been proposed (2)]. AP-5, similar to the other APs, comprises a core of four proteins, namely, ζ, β5, µ5 and σ5 subunits, which share structural similarity to corresponding subunits in other AP complexes. AP-5 is the most recently identified member of this protein family, and little is known about the cellular pathway(s) that AP-5 may play a role in, nor what the cargo specificity of AP-5 might be. However, some important clues are emerging from the integration of proteomics, cell biology and clinical genetics.

AP-5 has been shown to associate in a stable complex with two other proteins, spatacsin (SPG11) and spastizin [SPG15; FYVE-CENT; ZFYVE26 (3,4)], and to co-localize with markers of endosomes and lysosomes (4,5), suggesting a role of AP-5 along with spatacsin and spastizin in the endosomal pathway. Intriguingly, loss-of-function mutations in *SPG11* (6–9), *ZFYVE26* (SPG15) (10,11) and *AP5Z1* [SPG48 (3,12–14)] have all been described in patients with hereditary spastic paraplegia (HSP). HSPs are a group of neurological disorders typified by the degeneration of the long corticospinal axons leading to progressive lower limb muscle weakness and spasticity and further classified into pure or complex forms on the basis of additional neurological signs (15,16). Mutations in SPG11 and SPG15 generally result in a complex form of HSP, which is distinguished by prominent thinning of the corpus callosum, but also includes other neurological complications such as retinal abnormalities, intellectual disability, mild ataxia and parkinsonism (11,17). SPG48 patients have some clinical features similar to those of SPG11 or SPG15 patients, including spastic paraplegia, retinal abnormalities and parkinsonism, but the clinical spectrum of AP5Z1 patients is still being defined.

In this study, we investigate fibroblast lines from three patients harbouring distinct mutations in *AP5Z1*, which offers a unique framework to investigate where and how AP-5 may be functioning. We show that mutations in *AP5Z1* impair AP-5 complex formation and result in the accumulation of multilamellar structures containing aberrant storage material, revealing lysosomal dysfunction as the likely pathogenic mechanism.

## Results

### Effects of *AP5Z1* mutations on AP-5 protein abundance and localization

**Figure 1.**
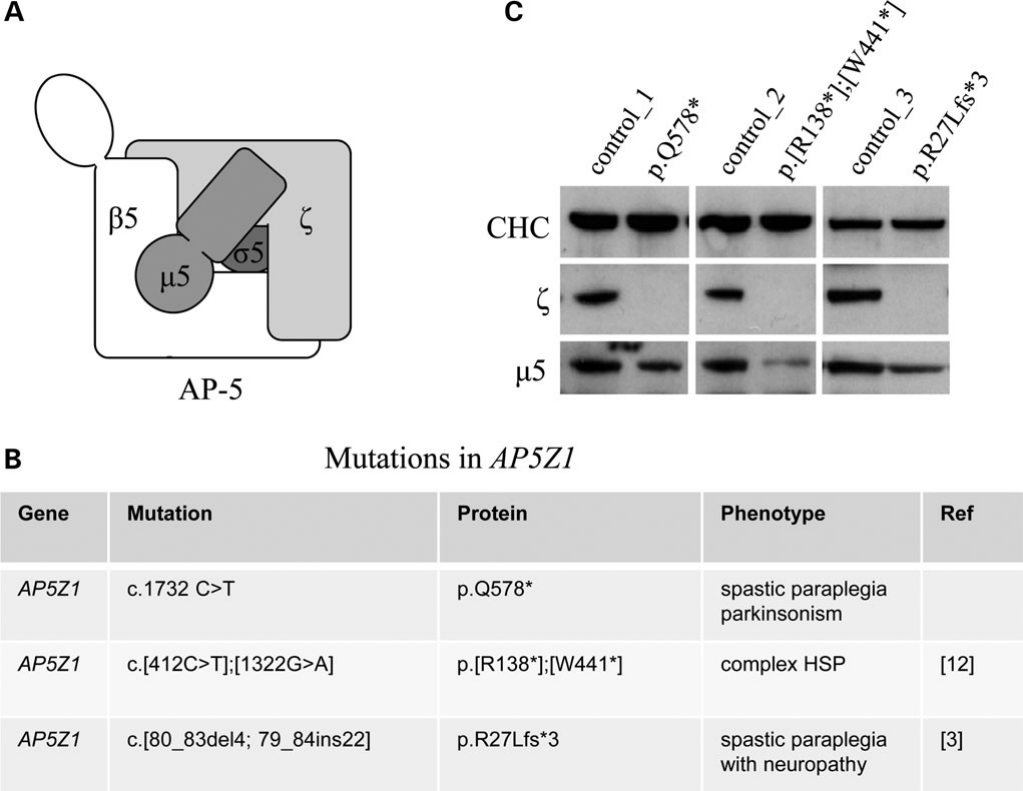
Effect of *AP5Z1* mutations on AP-5 protein expression. (**A**) Schematic diagram of AP-5 subunit organization. (**B**) Table detailing nonsense mutations in *AP5Z1* described in this study, in which premature terminations are indicated by asterisk. (**C**) Whole-cell western blots of patient-derived fibroblast lines including gender- and age-matched controls, loaded at equal protein levels and probed with antibodies against AP-5 ζ, AP-5 µ5 and clathrin (CHC; loading control). Note the loss of AP-5 ζ and concomitant reduction in levels of µ5.

AP-5 (Fig. 1A), similar to other AP complexes, is expressed in many tissues, including various regions of the brain and spinal cord, and at all stages of development (Supplementary Material, Fig. S1). This widespread expression pattern makes the use of fibroblasts derived from skin biopsies a relevant model system for the investigation of the cellular impact of mutations in *AP5Z1*. We analysed AP-5 protein levels in three SPG48 patient-derived fibroblast lines with mutations in *AP5Z1* : (i) c.1732C>T (p.Q578*), (ii) c.[412C>T];[1322G>A] (p.[R138*];[W441*]) and (iii) c.[80_83del4; 79_84ins22] (p.R27Lfs*3) (Fig. 1B), along with gender-matched and age-matched controls. The levels of AP-5 ζ protein correlated well with the predicted nature of the mutations, and AP-5 ζ protein was decreased to undetectable levels (Fig. 1C). In addition, there was a concomitant reduction in levels of µ5 compared with controls, which is due to protein instability of AP subunits that occurs in the absence of complex assembly (18–20). These results suggest that in these patient lines there is a loss of functional AP-5 and supports the obligate nature of AP complexes.

### Phenotypic effects of loss of AP-5

**Figure 2.**
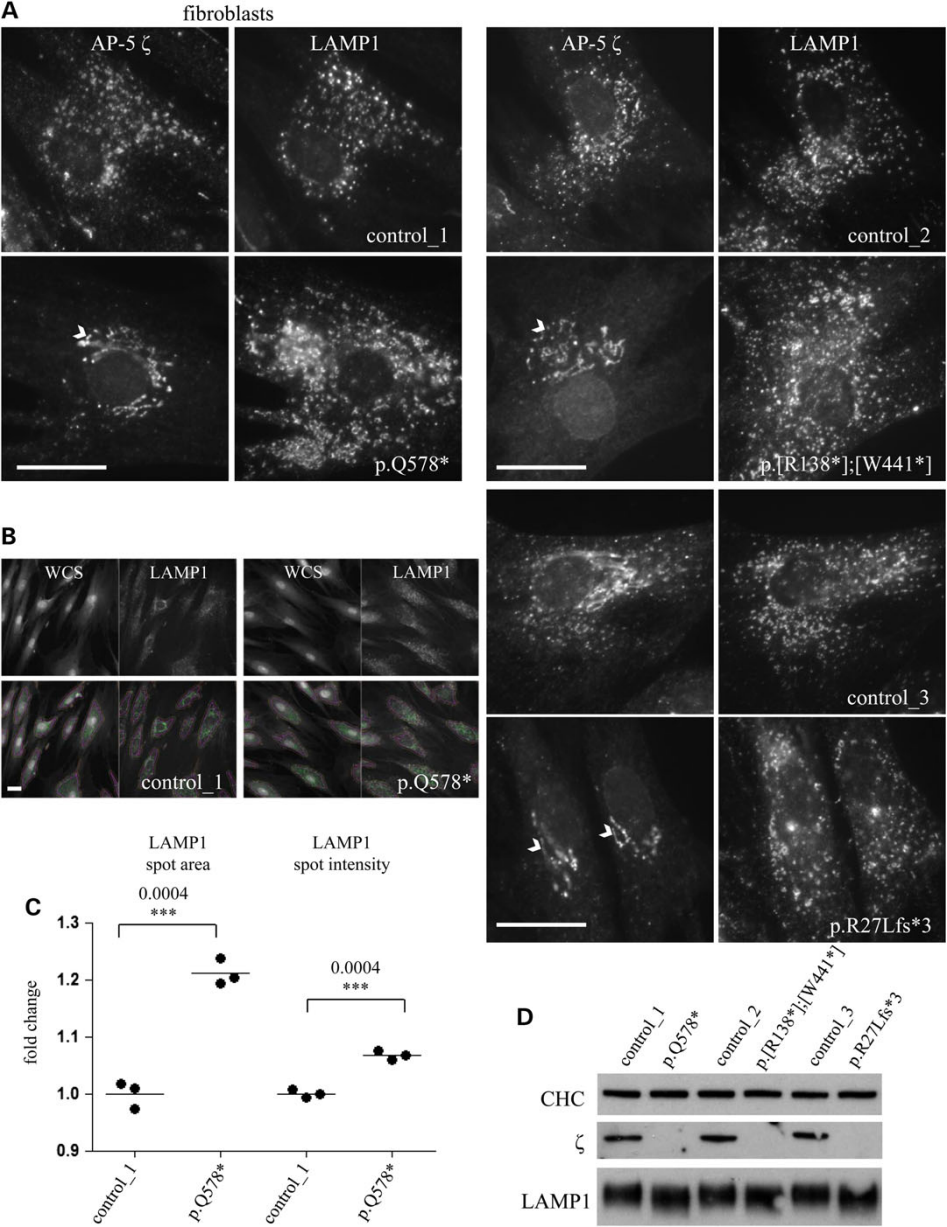
Loss of AP-5 in patient-derived fibroblasts. (**A**) Immunofluorescence of patient-derived fibroblasts (denoted by their mutation) and their respective gender- and age-matched controls doubled-labelled with antibodies against AP-5 ζ and LAMP1. Note that in control cells there is a significant overlap between LAMP1 and AP-5 ζ. In AP5Z1 patient lines, there is loss of the punctate AP-5 labelling pattern [the antibody cross-reacts with a reticular Golgi protein (white arrowhead), and this immunoreactivity persists when cells are depleted of AP-5 ζ by siRNA knockdown (4)]. Note that with the loss of AP-5 ζ, there is a consistent increase in the brightness and size of the LAMP1-positive puncta. Scale bar = 20 µm. (**B**) Patient-derived fibroblasts (p.Q578*) and their control (control_1) were plated onto 96-well plates and labelled with a whole-cell stain (WCS) and an antibody against LAMP1; the WCS allowed a mask to be drawn around the cells (pink) and various parameters of LAMP1 puncta (green) to be quantified on an ArrayScan Cellomics microscope using a SpotDetector algorithm. Scale bar = 20 µm (**C**) Spot average intensity and average area per object were quantified in three independent experiments, with more than 1000 cells quantified per experiment. Note that there is a subtle but significant increase in the area of the LAMP1-positive puncta. (**D**) Western blots of patient-derived fibroblasts probed for LAMP1, AP-5 ζ and clathrin (CHC; loading control). Note that there is no apparent increase in the total levels of LAMP1 protein.

In control fibroblasts, AP-5 ζ localized in fine puncta throughout the cytoplasm and co-localized with LAMP1, a marker of late endosomes and lysosomes (Fig. 2A, control lines). This is consistent with our previous localizations of AP-5 (4,21). In contrast, the AP-5 ζ punctate labelling was lost in all patient lines [note that the Golgi pattern seen here is due to a cross-reacting activity also noted in (4)]. The loss of AP-5 ζ correlated with the consistent appearance of larger LAMP1-positive puncta. Using automated microscopy, we could quantify an increase in the area of LAMP1 fluorescence (Fig. 2B and C), although this had no significant effect on total LAMP1 protein levels (Fig. 2D).

**Figure 3.**
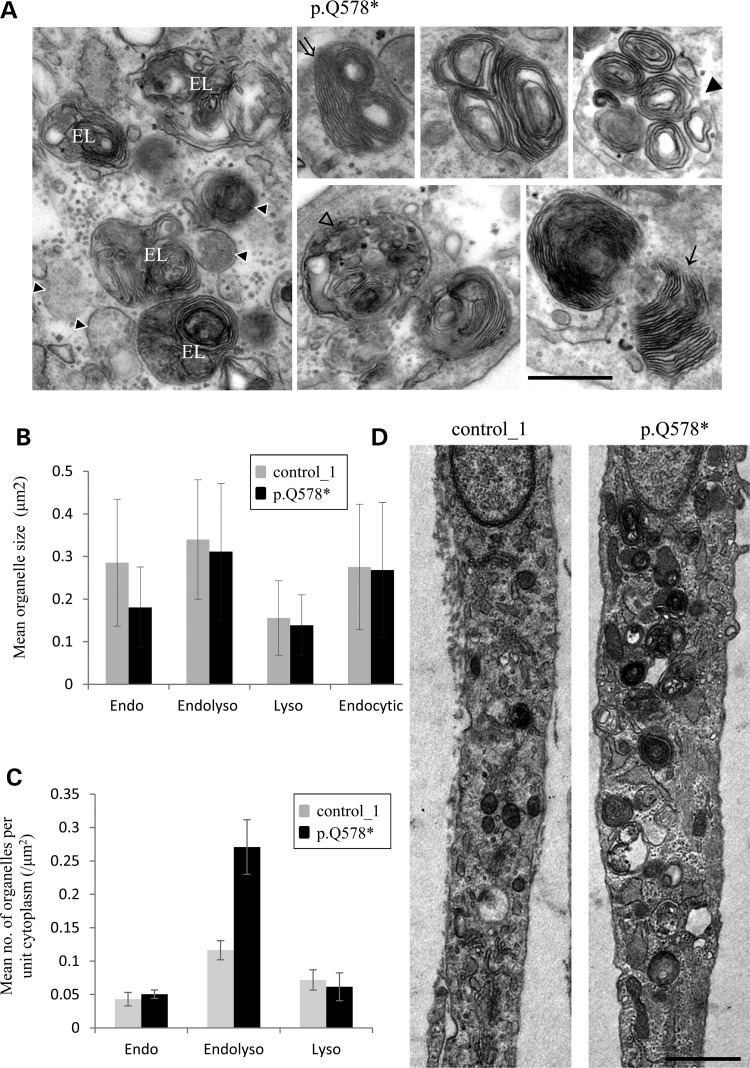
Ultrastructure of *AP5Z1* p.Q578* fibroblasts. (**A**) EM of patient-derived fibroblasts. Note the accumulation of morphologically defined endocytic structures filled with aberrant storage material typified by multiple exaggerated membrane whorls (filled arrow head), belts of striated material (double arrow), fingerprint bodies (arrow) and some intraluminal vesicles (open arrow head). These structures are predominantly surrounded by a single bilayer membrane, and there is clustering of endocytic structures marked by arrowheads with white outline (far left panel; EL, endolysosome). Scale bar = 500 nm. (**B**) Images were collected from 10 cells and the size of endocytic structures measured (96 for control_1 and 191 for the p.Q578* line); the individual data are shown for endolysosomes (Endolyso), endosomes (Endo) and lysosomes (Lyso) and combined together to show all endocytic structures. Note that no significant difference in the overall size of endocytic structures was revealed. (**C**) The number of different endocytic structures per unit area of cytoplasm was measured. Images were collected from 10 cells and the number of endocytic structures measured (96 for control_1 and 191 for the p.Q578* line). Note the increase in the number of endolysosomes per unit area of cytoplasm in the p.Q578* line. (**D**) Examples of *AP5Z1* p.Q578* fibroblasts showing the clustering of endolysosomes compared with its control. Scale bar = 1 µm.

Each patient-derived cell line was then assessed for morphological changes by electron microscopy (EM). Consistent with the immunofluorescence data, we observed large multilamellar structures surrounded by a single bilayer membrane (Fig. 3A and Supplementary Material, Fig. S2). These multilamellar structures had morphological features of hybrid organelles (derived from the fusion of endosomes and lysosomes), but were filled with aberrant storage material typified by multiple exaggerated whorls, belts of striations, fingerprint bodies and some intraluminal vesicles. Quantification of all morphologically defined endocytic structures showed that although the average overall sizes of endocytic structures had not changed significantly (Fig. 3B), there was a dramatic increase in the number of structures with features consistent with hybrid organelles (Fig. 3C and Supplementary Material, Fig. S2). It was also noted that there was a striking clustering of these multilamellar structures (Fig. 3D), and this likely accounts for the larger appearance of the LAMP1 immunolabelling in AP-5-deficient fibroblasts (Fig. 2A) and the increase in the area of LAMP1 puncta quantified for the p.Q578* line on the automated microscope (Fig. 2C). Generally, by EM, the control fibroblasts contained morphologically ‘normal’ endosomes and lysosomes, and only occasionally contained some storage material that appeared as a belt of striations or as a single whorl, but much less exaggerated in appearance and far less common than in patient fibroblasts (Fig. 3D and Supplementary Material, Fig. S2).

**Figure 4.**
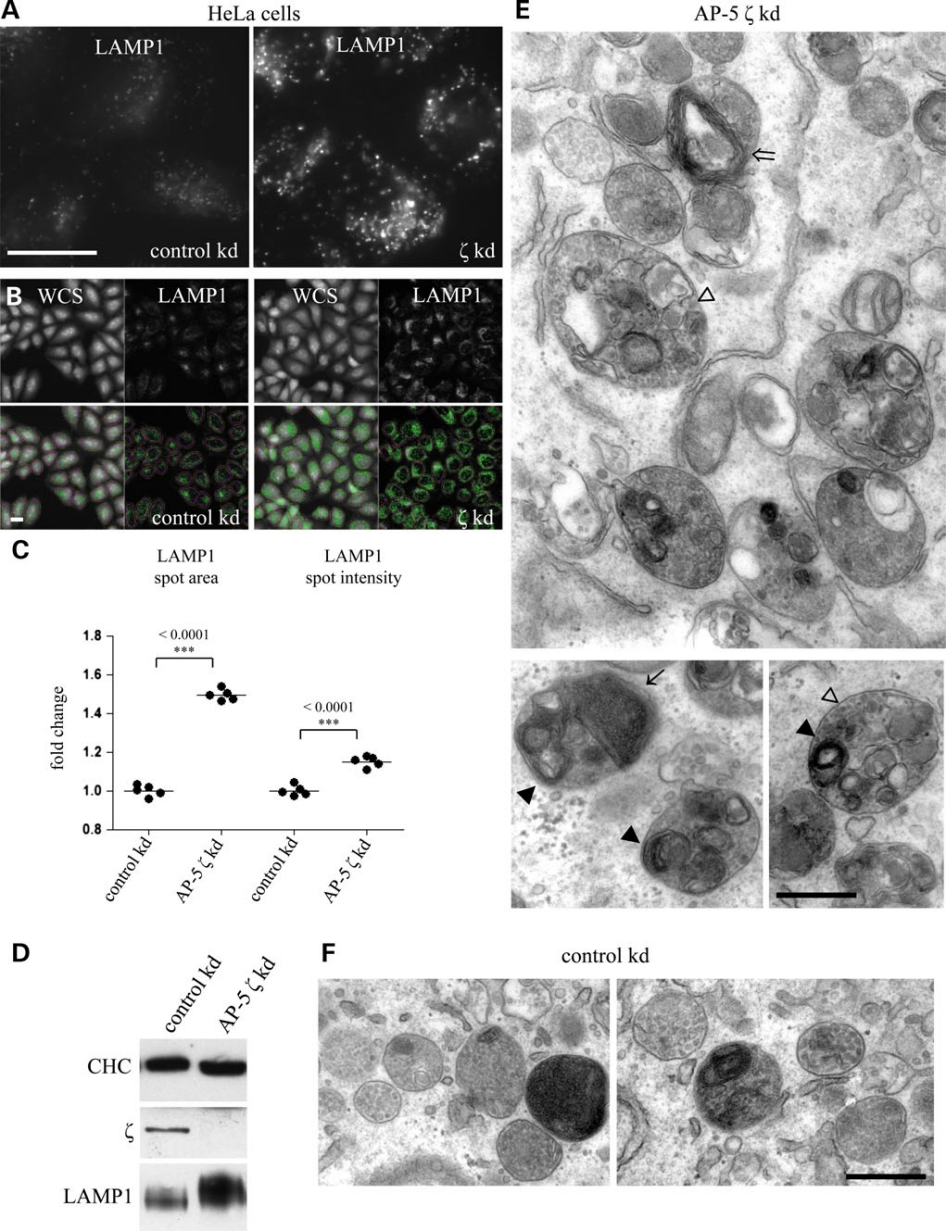
Loss of AP-5 by siRNA knockdown in HeLa cells. (**A**) Immunofluorescence of HeLa cells treated with siRNA targeting AP-5 ζ or non-targeting control labelled with an antibody against LAMP1. Note that the depletion of AP-5 ζ results in brighter and larger LAMP1 puncta. Scale bar = 20 µm. (**B**) HeLa cells treated with an siRNA targeting AP-5 ζ or non-targeting control were plated onto 96-well plates and labelled with a whole-cell stain (WCS) and an antibody against LAMP1; the WCS allowed a mask to be drawn around the cells (pink) and various parameters of LAMP1 labelling (green) to be quantified on an ArrayScan Cellomics microscope using a SpotDetector algorithm. Scale bar = 20 µm. (**C**) Spot average intensity and average area per object were quantified in three independent experiments, with more than 1000 cells quantified per experiment. Note the significant increase in the area of LAMP1-positive puncta. (**D**) Western blots of HeLa cells treated with an siRNA targeting AP-5 ζ or non-targeting control were loaded at equal protein and probed with antibodies against LAMP1, AP-5 ζ and clathrin (CHC; used as a loading control). Note that there is an increase in the total level of LAMP1. (**E**) EM was performed on HeLa cells treated with siRNA targeting AP-5 ζ (E) or a non-targeting control (**F**). Scale bar = 500 nm. Note that the loss of AP-5 ζ led to the accumulation of enlarged morphologically defined endosomal structures filled with aberrant storage material, typically containing bands of striated material, multiple exaggerated membrane whorls and many intraluminal vesicles.

In HeLa cells knocked down using siRNA against AP-5 ζ, we could recapitulate the appearance of larger LAMP1 puncta (Fig. 4A) and could confirm this using an alternative marker of late endocytic and lysosomal structures, CD63 (Supplementary Material, Fig. S3). We could quantify an increase in brightness and area of LAMP1 labelling on an automated microscope (Fig. 4B and C), which was reflected in an increase in the overall levels of LAMP1 (Fig. 4D). In general, these effects were more striking than those in the patient fibroblasts (Fig. 2C), which could be explained by the heterogeneity in the size and shape of patient fibroblasts that makes quantification of differences harder to measure and/or reflects the difference in acute versus chronic knockdown (i.e. patient cells may have had time to engage compensatory mechanisms). Consistent with the patient fibroblast EM data, ultrastructural analysis revealed the accumulation of multilamellar structures with morphological features of hybrid organelles, along with an array of aberrant storage materials (compare Fig. 4E with F).

Collectively, these observations indicate that the accumulation of enlarged late endolysosomal structures containing aberrant storage material is due to the loss of AP-5. Furthermore, the accumulation of aberrant storage material is reminiscent of features described in many lysosomal storage diseases (LSDs) and is indicative of dysfunction along the endosomal/lysosomal pathway.

### Relationship between spatacsin/spastizin and AP-5

**Figure 5.**
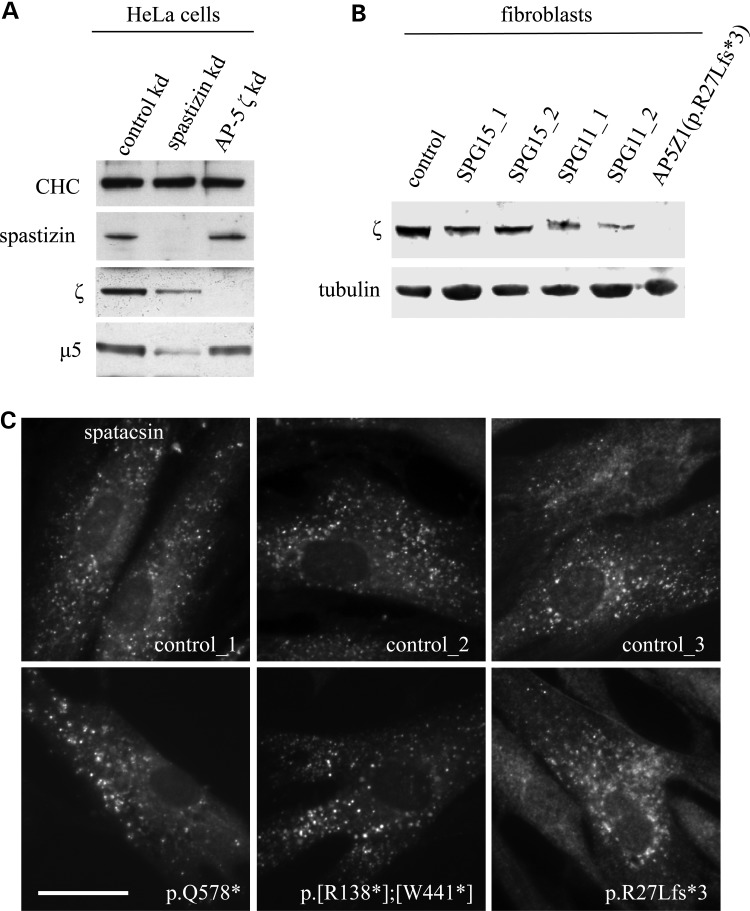
AP-5 is dependent on spatacsin and spastizin. (**A**) Western blots of HeLa cells were treated with siRNA against spastizin or non-targeting control and probed with antibodies against spastizin, AP-5 ζ and clathrin (CHC; used as a loading control). Note that there is loss of AP-5 ζ and µ5 when spastizin is depleted. (**B**) Western blots of patient fibroblasts with mutations in *AP5Z1* (Patient 3: p.R27Lfs*3), *SPG11* or *SPG15* were probed with antibodies against AP-5 ζ and tubulin (used as a loading control). Note that there is loss of AP-5 ζ in SPG11 and SPG15 patient fibroblasts. (**C**) Immunofluorescence of patient-derived fibroblast cells and controls were labelled with antibodies against spatacsin. Note that in patient cells lacking AP-5 ζ, there is no significant loss of membrane-associated spatacsin, and the puncta appear larger. Scale bar = 20 µm.

Although spatacsin and spastizin have been shown to associate with AP-5 (3,4), there are conflicting reports about the function of spastizin (3,22–24), as well as the obligate nature of their association (5,25). Here we qualify their relationship and show that depletion of spastizin using siRNA in HeLa cells leads to reduction of the protein levels of both AP-5 ζ and µ5 (Fig. 5A). We also find that there is a consequent accumulation of aberrant LAMP1-positive structures (Supplementary Material, Fig. S4), reminiscent of what has been previously reported in the SPG15 knockout mouse (5), patient fibroblasts (26) and HeLa knockdown cells (25). Consistent with these observations, we also find a reduction in the AP-5 ζ protein levels in SPG11 and SPG15 patient fibroblasts (Fig. 5B). The reciprocal relationship, however, does not appear to be the same, with depletion of AP-5 ζ not altering the total level of spastizin (Fig. 5A) or the membrane association of spatacsin (Fig. 5C). Taken together, we find that although AP-5 is dependent on spastizin and spatacsin, it is possible that spatacsin and spastizin can function independently of AP-5.

### Loss of AP-5 mutations leads to accumulation of endolysosomes

The maintenance of endosomal and lysosomal homeostasis is complex, and progress through the endocytic pathway involves homotypic fusion between endosomes as well as heterotypic fusion between endosomes and lysosomes (27,28). Endolysosomes are defined as hybrid or intermediate compartments that appear transiently, following the fusion of endosome and lysosome [prior to reformation of lysosomes (28–30)], and are hydrolytically active (29).

**Figure 6.**
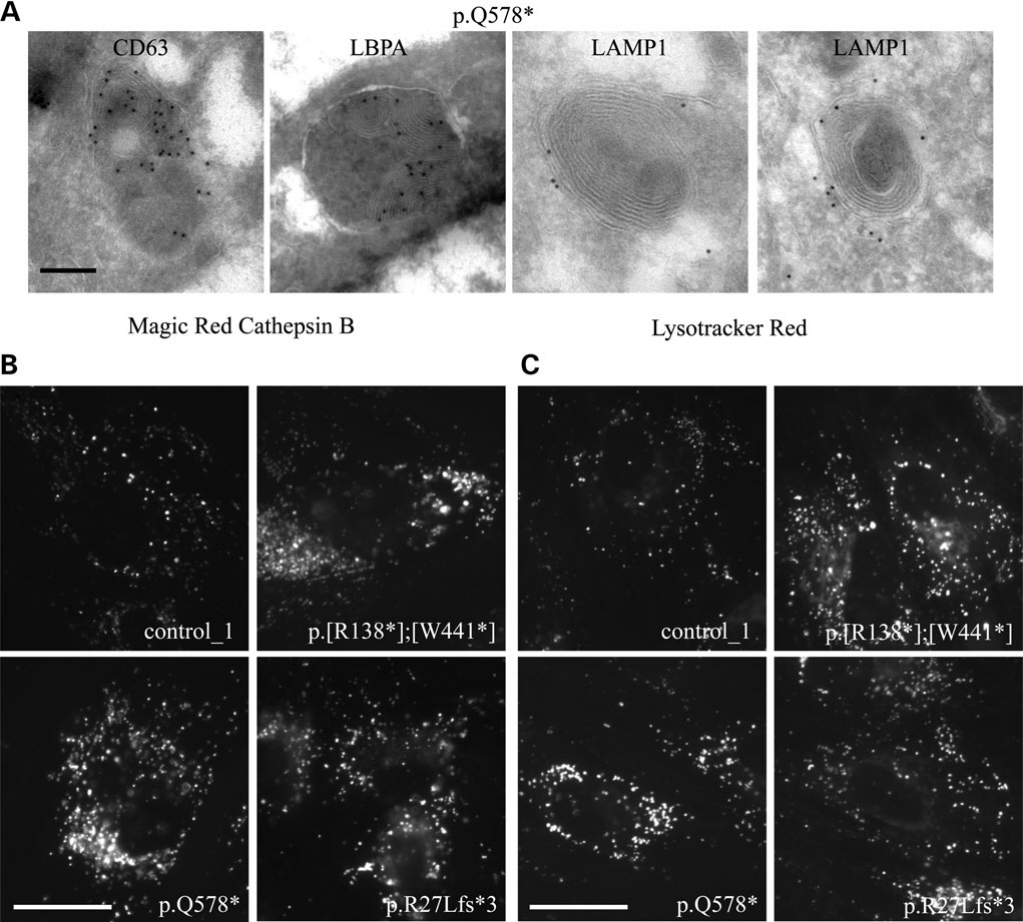
Identification of endolysosomes in AP-5 patient lines. (**A**) Cryo-immunoelectron microscopy of patient-derived fibroblasts (p.Q578*) labelled with antibodies against the late endosomal/lysosomal markers, LAMP1, LBPA and CD63. Note that the enlarged endocytic structures label positive for all markers, with LAMP1 labelling restricted to the limiting membrane, and LBPA and CD63 largely restricted to intraluminal whorls and striations. Scale bar = 200 nm. (**B**) Live imaging of control and patient-derived fibroblasts following incubation with Magic Red Cathepsin B reagent for 45 min at 37°C. Note that the enlarged structures in the patient lines are positive for cathepsin B activity and are therefore hydrolytically active. Scale bar = 20 µm. (**C**) Live imaging of control and patient-derived fibroblasts incubated with Lysotracker Red at 37°C. Note that the enlarged structures in the patient lines are positive for Lysotracker Red and are therefore acidic. Scale bar = 20 µm.

In order to investigate further the nature of the enlarged multilamellar structures in the AP-5 patient lines, we used cryo-immunoelectron microscopy and acidotropic and hydrolytic-activity-dependent fluorescent probes. Correlating with the immunofluorescence, we observed enlarged multilamellar structures positive for LAMP1, CD63 and lysobisphosphatidic acid (LBPA) (Fig. 6A). Specifically, LAMP1 was restricted to the limiting membrane rather than internal membranes, whereas CD63 and LBPA, which are enriched on intraluminal vesicles (31,32), were principally associated with intraluminal whorls and striations.

We also labelled live cells with Lysotracker Red, a fluorescent dye for labelling and tracking acidic organelles in live cells, and Magic Red Cathepsin B substrate, which is used to assess cathepsin activity (31), and could show that these enlarged structures were acidic as well as hydrolytically active (Fig. 6B and C).

**Figure 7.**
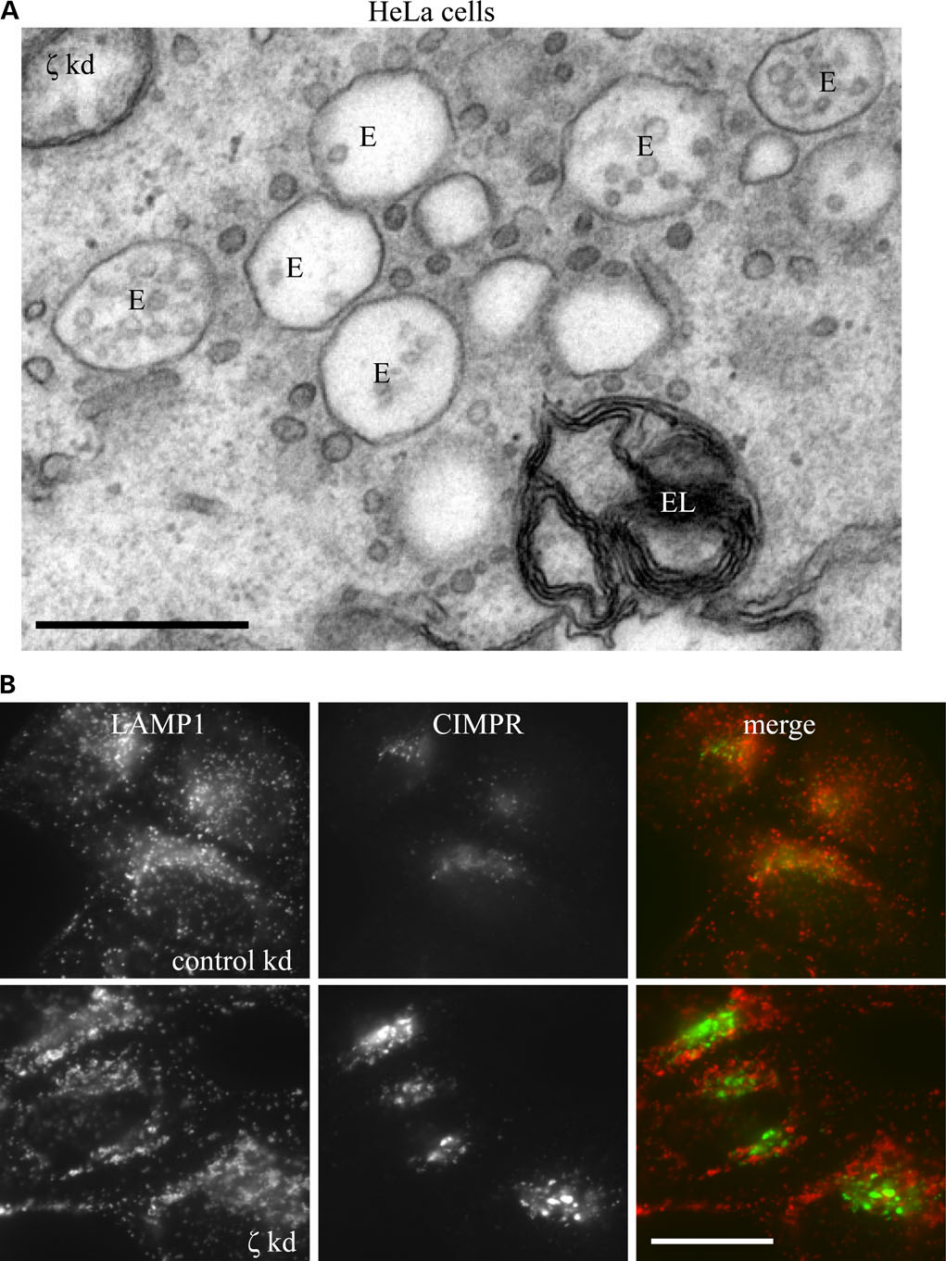
Loss of AP-5 results in the clustering of early endosomes. (**A**) EM of HeLa cells knocked down for AP-5 ζ. Note the clustering of morphologically defined early endosomes (typified by being electron-lucent with few intraluminal vesicles), surrounded by many small vesicles that we believe to be tubules in cross-section. Adjacent to this cluster of early endosomes is an example of an endolysosome filled with aberrant storage material. Scale bar = 500 nm. (**B**) Immunofluorescence of HeLa cells treated with siRNA against AP-5 ζ or a non-targeting control and double-labelled with antibodies against CIMPR and LAMP1. Note that the depletion of AP-5 ζ resulted in the clustering of CIMPR labelling that does not overlap significantly with LAMP1. Scale bar = 20 µm.

In HeLa cells depleted of AP-5 ζ, not only did we observe accumulation of aberrant multilamellar structures, but also clustering of morphologically defined early endosomes (Fig. 7A), which by immunofluorescence labelled positive for CIMPR and distinct from the LAMP1 labelling (Fig. 7B). This is reminiscent of the phenotype that we reported following the knockdown of any one of the subunits of AP-5, although in our previous study we did not look at the impact of loss of AP-5 on LAMP1 (21). We could not do equivalent experiments in fibroblast lines as at steady state CIMPR is mainly localized to the trans-Golgi network (Supplementary Material, Fig. S5).

As AP-5 is known to associate with spastizin, which has been reported to play a role in either autophagosome–lysosome fusion (23) or recovery of lysosomes from autolysosomes (25), we felt it pertinent to determine whether these enlarged multilamellar structures were positive for LC3, a marker of autophagosomes. Under basal conditions, we found that these enlarged LAMP1-positive structures did not label for LC3 in either HeLa knockdowns (Supplementary Material, Fig. S6A) or patient fibroblasts (Supplementary Material, Fig. S6B), although there appears to be very little basal autophagy in these cells. This finding led us to believe that these structures were unlikely to be autophagosomes.

Collectively, these observations define these enlarged aberrant structures as having characteristics of endolysosomes and as such are likely to be the product of the fusion of late endosomes with lysosomes. Furthermore, the accumulation of these structures results in the clustering of early endosomes, which suggests that there is a disruption of endosomal and probably lysosomal dynamics/homeostasis.

## Discussion

Here we present the description of three patient-derived fibroblast lines with mutations in *AP5Z1*. Although two mutations have been reported previously (3,12), the impact of these mutations on AP-5 and at the cellular level had not been determined. All the patients presented with spastic paraplegia that was accompanied by neuropathy, parkinsonism and/or cognitive impairment (3,12). At the cellular level, these lines show loss of AP-5 ζ protein along with a reduction in levels of AP-5 µ5, consistent with these proteins existing as an obligate complex (4). Ultrastructural analysis of patient-derived lines and HeLa cells depleted of AP-5 ζ demonstrate that the loss of AP-5 results in an accumulation of enlarged multilamellar structures that are filled with aberrant storage material. Age does not appear to be a significant contributor to this phenotype as these features were not observed in age-matched controls, nor is there a strong correlation with severity and age.

AP-5 ζ and spastizin have been shown to associate in a stable complex, along with core members of AP-5 (β5, µ5 and σ5) and spatacsin (3,4). In agreement with this observation, the subclinical loss of function of both spatacsin and spastizin was associated with abnormal motoneuron development in zebrafish (33). This provided a molecular explanation for the close similarity in the clinical presentation of SPG11 or SPG15 (17) and their overlap with SPG48 patient features (3,12,13). What can the cell biology characterization tell us about the relationship between AP-5 and spatacsin/spastizin? Here we show that abrogation of spatacsin and spastizin results in the decrease of AP-5, similar to results in SPG11 or SPG15 patient-derived fibroblasts (25) and HeLa knockdown studies (4). Collectively, these observations suggest that AP-5 is dependent on spatacsin and spastizin for either its assembly or its recruitment to membranes, but the converse is not true as spatacsin and spastizin are still recruited to membrane in the absence of AP-5 (see also 4,25). The fact that spatacsin and spastizin may be able to function in the absence of AP-5 may help explain alternative functions attributed to SPG15, such as in dense core granule formation (22), or its role in autophagosome fusion (23).

What is the nature of the aberrant multilamellar structures found in patients with mutations in *AP5Z1*? We find that they label for LAMP1 at the limiting membrane, and CD63 and LBPA are largely restricted to intraluminal membrane whorls; they are acidic and hydrolytically active, are not positive for the autophagy marker LC3 and are surrounded by a single bilayer membrane. In the context of current models, these features are consistent with endolysosomes, and we would suggest that the storage material results from the partial degradation of intraluminal vesicles that are CD63- and LBPA-positive (31,32). Notably, similar ultrastructural features have been described for several LSDs including metachromatic leukodystrophy, Fabry disease, mucopolysaccharidoses, Niemann–Pick disease, GM2 gangliosidoses, I-cell disease [where EM features have been reported as lamellar lipids and ‘zebra bodies’ (34)], mucolipidosis (‘concentric multilaminar membrane structures’ (35)] and neuronal ceroid lipofuscinosis [‘fingerprint bodies’ (36,37)].

Does AP-5 deficiency define a new type of LSD? Although LSDs were first classified on the basis of lysosomal enzyme deficiency that leads to inability to metabolize lipids, glycoproteins or glycosaminoglycans, there are now many LSDs that include deficiencies in lysosomal transmembrane proteins, soluble non-enzymatic lysosomal proteins and proteins important for trafficking to or from lysosomes (34,38–40). The broader definition of LSDs therefore encompasses defects in the lysosomal structure and function (41) that, for less well-defined reasons, result in the accumulation of undegradable material in the endosomal/lysosomal system. Two classes of LSDs have been proposed: primary LSDs that arise from defects in specific lysosomal enzymes and secondary LSDs that result from impaired membrane traffic (42). Therefore, although at this point it is not clear what causes the accumulation of storage material in AP-5-deficient fibroblasts, it seems likely to be a secondary LSD, and the exaggerated membrane whorls could point to a deficit in the ability to metabolize lipids (34). Interestingly, the clinical features of neuropathic LSDs are similar to those described in SPG48 patients (3,12,13), as well as SPG11 and SPG15, including cognitive impairment, ataxia, peripheral neuropathy, parkinsonism and spasticity (42). Clinically, it can be challenging to distinguish classical LSDs and some forms of complex HSP, as evidenced by reports of patients originally suspected to have HSP but subsequently diagnosed with LSD [e.g. Krabbe's disease (43) and ceroid lipofuscinosis (14)].

For many LSDs, it has been reported that the progressive accumulation of storage material leads to the build-up of enlarged (>1 µm) lysosomes that are dysfunctional (44). The parallels between loss of AP-5 and LSDs allow us to hypothesize a simple model for the pathogenesis of AP-5 deficiency related to lysosomal dysfunction. Here, we find that AP-5 is expressed in neurons of the cortex, hippocampus and cerebellum, as well as in non-neuronal tissues. Neurons are known to be particularly sensitive to aberrant lysosomal storage or dysfunction. The questions of what the increase in endolysosome frequency means, why aberrant storage material accumulates, how this impacts neuron survival and what this means for the role of AP-5 are complex. The increase in endolysosome frequency may arise by a number of scenarios that are not mutually exclusive. These include increased endosome and lysosome fusion events, increased biosynthetic delivery [for example, by LAMP carriers (45)], decreased degradation of content or decreased lysosomal recovery. In addition, the accumulation of endolysosomes will likely impact upon upstream and downstream endosomal and lysosomal functions, as well as other organelle functions (42). Indeed, we have seen that in HeLa cells depleted of AP-5 ζ, CIMPR accumulates in early endosomes, which may underlie a more general perturbation of membrane trafficking, and could be due to the displacement of fusion machinery (e.g. SNAREs).

In the context of current models, our data are consistent with the loss of AP-5 disturbing the equilibrium between endosomes, endolysosomes and lysosomes. We favour a model whereby AP-5 could act in the recovery of lysosomes from endolysosomes because of its association with spatacsin and spastizin and the mechanistic similarities with lysosome reformation from autolysosomes (25). This is in keeping with the role of other LSD proteins including TRPML1 [mucolipidosis type IV (46)] and NPC2 [Niemann–Pick type C (47)] that are involved in the regulation of lysosome formation. Given the role of AP complexes in protein sorting, it seems plausible that the main role of AP-5 may be in the selection of cargos to/from these organelles, the loss of which impacts upon endosome/lysosome homeostasis. One of the challenges for the future will be to identify possible AP-5 cargo proteins (as well as accessory proteins) through the use of technologies such as CRISPR knockout and acute ablation techniques. This may help define not only the role of AP-5 in normal cell physiology but may clarify how loss of function of AP-5 may lead lysosomal dysfunction and to disease. This in turn may be relevant to other HSPs (particularly SPG11 and SPG15) and LSDs and expand our understanding of recessive HSPs. Furthermore, it will be interesting to determine whether there are mutations in the other subunits of AP-5 and to determine the full spectrum of clinical features. Although the number of HSP patients with mutations in *AP5Z1* is still low, their description reaffirms the important roles of all five AP complexes, which is encompassed by the broad definition of ‘adaptinopathy’ that was coined to define this class of disorders of intracellular trafficking (2).

## Materials and Methods

### Patients and patient-derived fibroblasts

Patient p.Q578* is a man in his early 70s (age of symptom onset about 60) with neuropathy, spastic paraplegia, ataxia, retinopathy and parkinsonism. Patient p.[R138*];[W441*] is a woman in her 50s (age of onset late 40s), with a complex form of HSP with cognitive decline (12). Patient p.R27Lfs*3 is a woman in her 80s who initially presented with urinary incontinence at 49 years of age, which progressed to spastic paraplegia and neuropathy (3). Fibroblasts were established from skin punch biopsies from these patients along with gender- and age-matched controls, under approved clinical protocols (NINDS Institutional Review Board, protocol 2000-N-0043), with subjects' informed consent obtained. Following establishment of patient lines and controls, all patient and control cells were cultured side-by-side, and experiments were performed on equivalent passage number (all less than 10 passages) in order to reduce operator differences and differences due to time in culture.

For comparison, we also used fibroblasts from two SPG11 and two SPG15 patients with symptoms typical of these pathologies. One SPG15 patient of Portuguese origin carries the homozygous c.6296dup (p.N2100Efs*12) mutation (48) and another from Syria carries the homozygous c.5036delT (p.L1679Rfs*1687) mutation (10). One SPG11 patient from Morocco carries the homozygous c.6100C>T (p.Arg2034*) nonsense variant (6), and the second SPG11 case originated from Latvia and has a heterozygous c.2431C>T (p.Q811*) variant in *trans* of a deletion affecting exon 29 (A.D. and G.S., unpublished data).

### Antibodies

Antibodies used in this study include in-house antibodies against clathrin, AP-5 ζ (monoclonal antibody used for IF), µ5 and SPG11 monoclonal (4) and spastizin [PER antibody (49)] and commercial antibodies against spastizin (Atlas HPA035693), EEA1 (BD Transduction Labs E41120), LAMP1 (Abcam ab24170 and H4A3), CIMPR (2G11; Calbiochem 444105), AP-2 µ2 (AP50; BD Transduction Labs 611351), LC3 (4E12; MBL M152-3B), LBPA (Jean Gruenberg), CD63 (H5C6; BD Biosciences), tubulin (DM1A, Abcam ab7291) and actin (C4, Abcam ab3280). Horse radish peroxidase-labelled secondary antibodies were purchased from Sigma and fluorescently labelled secondary antibodies from Invitrogen.

### Tissue culture and knockdowns

Patient-derived fibroblasts and HeLa M cells were grown in Dulbecco's modified Eagle's medium (DMEM, Sigma), supplemented with 10% (v/v) fetal calf serum (Sigma), 2 mm l-glutamine, 50 U/ml penicillin and 50 µg/ml streptomycin. Knockdowns in HeLa cells were performed using the following On-Target Plus SMARTpool siRNA reagents from Dharmacon, with a non-targeting Smartpool siRNA (D-001810-10) used as a control. The siRNAs were: AP-5 ζ (KIAA0415), L-025284-01 and SPG15: L-031136-01 and were deconvoluted as in (4). All siRNAs were used at a concentration of 25 nm, and knockdowns were performed with a double-hit 96 h protocol using Oligofectamine (Invitrogen) and Opti-MEM, following the manufacturer's instructions.

### Western blotting and tissue preparation

To analyse tissue distribution of AP-5, a mouse or rat was decapitated and tissues were harvested and homogenized with a dounce homogenizer in lysis buffer (10 mm Tris–HCl pH 7.4, 100 mm NaCl, 1 mm EGTA, 2 mm MgCl_2_, 1% sodium dodecyl sulphate) containing complete protease inhibitor (Roche) and 10 U/ml Benzonase (Novagen). Protein concentrations were quantified with BCA protein assay kit (Pierce). Western blotting was performed as described previously (49).

### *In situ* hybridization

Two-month-old rats were killed by decapitation and brains rapidly extracted and fixed in 4% paraformaldehyde for 48 h. Samples were transferred into phosphate-buffered saline (PBS) containing 30% sucrose for 48 h and then frozen in isopentane (−30°C). The *in situ* hybridization was performed on 20 µm coronal cryosections as described previously (5), using digoxigenin-labelled sense or antisense riboprobes at a concentration of 100 ng/µl. The RNA probes covered cDNA positions 15–482 and 1940–2413 of rat *AP5Z1* transcript (accession number NM_001037220.4).

### Immunofluorescence and quantification

For immunofluorescence microscopy, cells were plated into glass-bottom dishes (MatTek). The cells were then fixed with 3% formaldehyde, permeabilized with PBS/0.1% Triton X-100/0.5% bovine serum albumin (BSA) or −20°C methanol for 5 min and then blocked with PBS/0.5% BSA. Cells were labelled as indicated and imaged with a Zeiss Axiovert 200 inverted microscope using a Zeiss Plan Achromat 63× oil immersion objective (NA 1.4), a Hamamatsu OCRA-ER2 camera and Improvision Openlab software.

To quantify knockdown phenotypes, we used an automated ArrayScan VTI microscope (Cellomics/Thermo-Fisher) and the SpotDetector V4 assay algorithm. Cells were plated onto 96-well Perkin Elmer microplates, fixed then stained with antibodies against CD63 or LAMP1, followed by Alexa Fluor 488-donkey anti-mouse IgG, blue whole-cell stain (Invitrogen) and far red beads to assist in sample focusing (Flow-Check fluorospheres; Beckman Coulter). The cells were imaged with a modified Zeiss Axiovert 200M inverted microscope, a Zeiss 40×/0.5 Achroplan objective and a Hamamatsu OCRA-ER camera. More than 1000 cells were quantified for each condition using ARRAYSCAN software.

LysoTracker^®^ Red DND-99 (Life Technologies) is a red-fluorescent dye that is membrane-permeable and specifically accumulates in acidified compartments. Cells were plated onto glass-bottom dishes and incubated with 50 nm Lysotracker Red and imaged immediately live. Magic Red Cathepsin B substrate (AbD Serotec) is a membrane-permeable probe which becomes fluorescent upon cleavage by cathepsin B. Cells were plated onto glass-bottom dishes and incubated in CO_2_-independent media for 45 min at 37°C using Magic Red substrate, according to manufacturer's instructions, and then visualized live.

### Conventional EM

Cells were grown on plastic dishes (fibroblasts were left to recover in culture for 7 days following trypsinization) and fixed using double-strength fixative [4% paraformaldehyde (PFA), 5% glutaraldehyde, 0.1 m cacodylate buffer pH 7.2], added to an equal volume of culture media. After 2 min, double-strength fix was replaced by single-strength fix and cells were fixed for 1 h. Cells were then scraped and pelleted. Cells were then secondarily fixed with 1% osmium tetroxide and incubated with 1% tannic acid to enhance contrast. Cells were dehydrated using increasing percentages of ethanol before being embedded in EPON in beam capsules. EPON was cured overnight at 65°C.

Ultrathin (50–70 nm) conventional sections were cut using a diamond knife mounted to a Reichart Ultracut S ultramicrotome. Sections were collected onto copper grids. Grids were stained using lead citrate. Sections were viewed on an FEI Tecnai transmission electron microscope (Eindhoven, The Netherlands) at 80 kV.

The number and size of different endocytic structures per unit area of cytoplasm were quantified. For each cell line and respective age-matched control, complete cell montages from 10 cells were acquired. Total cytoplasmic volumes were calculated using ImageJ and the number of organelles scored on the basis of morphology; where endosomes were defined as having an electron lucent lumen, may contain intraluminal vesicles, but no membrane whorls, and endolysosomes as containing membrane whorls, may contain intraluminal vesicles and a lucent or dark lumen and lysosomes having an electron dark lumen and no membrane whorls or intraluminal vesicles. Organelle sizes were measured using ImageJ. Means and standard errors are shown.

### Cryo-immunoelectron microscopy

For immunogold labelling, fibroblasts were fixed by adding an equal volume of freshly prepared 4% PFA/0.2% glutaraldehyde in 0.1 m phosphate buffer, pH 7.4. After 2 min, the solution was removed and cells were post-fixed in 2% PFA/0.1% glutaraldehyde in 0.1 m phosphate buffer, pH 7.4, for 2 h at room temperature. Cells were scraped, pelleted and embedded in 12% gelatin at 37◦C and infused with 1.7 m sucrose/15% polyvinyl pyrolidone overnight at 4◦C before being mounted to stubs in liquid nitrogen. Ultrathin frozen sections were cut with a diamond knife in a Reichert Ultracut S ultramicrotome (Leica, Germany), collected with either 2% methylcellulose: 2.3 m sucrose (1:1 ratio) or 2.3 m sucrose alone and mounted on Formvar-coated grids. Ultrathin sections were labelled with primary antibodies and detected using protein-A conjugated to 10 nm colloidal gold (Utrecht University, The Netherlands). Sections were viewed on an FEI Tecnai transmission electron microscope (Eindhoven) at 80 kV.

## Supplementary Material

Supplementary Material is available at *HMG* online.

## Funding

This work was supported by the Wellcome Trust (086598; J.H., J.R.E. and M.S.R.), the European Union (OMICS Call, Neuromics project; F.D., A.D., T.E. and G.S.), Verum Foundation (G.S.), Programme d'Investissement d'Avenir (ANR-10-IAIHU-06; F.D., A.D., T.E. and G.S.), ERC starting grant (311149; F.D.), the Intramural Research Program of the NINDS, National Institutes of Health (J.C., R.H.R. and C.B.) and National Institutes of Health grant NS083739 (M.M. and M.C.K.), Doris Duke Foundation (Clinical Scientist Development Award to M.C.K.) and Healthcare Research of the Italian Ministry of Health (C.M. and C.G.). C.G. also received partial research support by AriSLA (NOVALS 2010). Funding to pay the Open Access publication charges for this article was provided by the Wellcome Trust.

## Supplementary Material

Supplementary Data
