# Supplementary material for: Loss of AP-5 results in accumulation of aberrant endolysosomes: defining a new type of lysosomal storage disease
Source: Hum Mol Genet. 2015 Jun 17;24(17):4984–96. doi: 10.1093/hmg/ddv220 (PMC4527494; doi:10.1093/hmg/ddv220)
Supplement: Supplementary Data [file supp_24_17_4984__index.html]

Loss of AP-5 Results in Accumulation of Aberrant Endolysosomes, Defining a New Type of Lysosomal Storage Disease — Loss of AP-5 results in accumulation of aberrant endolysosomes: defining a new type of lysosomal storage disease — Loss of AP-5 results in accumulation of aberrant endolysosomes: defining a new type of lysosomal storage disease — Supplementary Data 

# Loss of AP-5 results in accumulation of aberrant endolysosomes: defining a new type of lysosomal storage disease

## Supplementary Data

Supplementary Data

- Supplementary Figures - pdf file
- Supplementary Data - Doc file
