# Supplementary figures and images for: Loss of AP-5 results in accumulation of aberrant endolysosomes: defining a new type of lysosomal storage disease
Source: Hum Mol Genet. 2015 Jun 17;24(17):4984–96. doi: 10.1093/hmg/ddv220 (PMC4527494; doi:10.1093/hmg/ddv220)

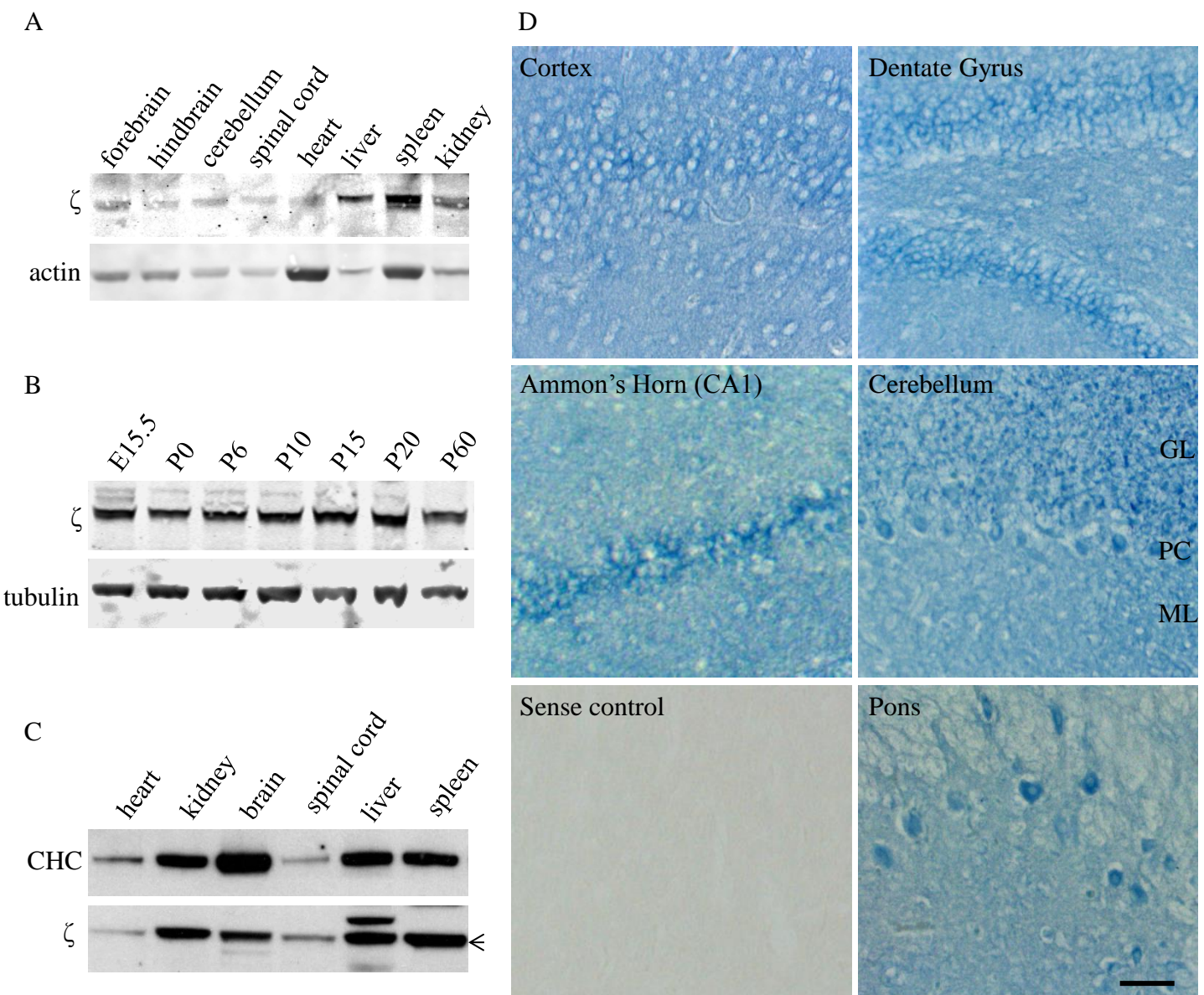

Figure S1

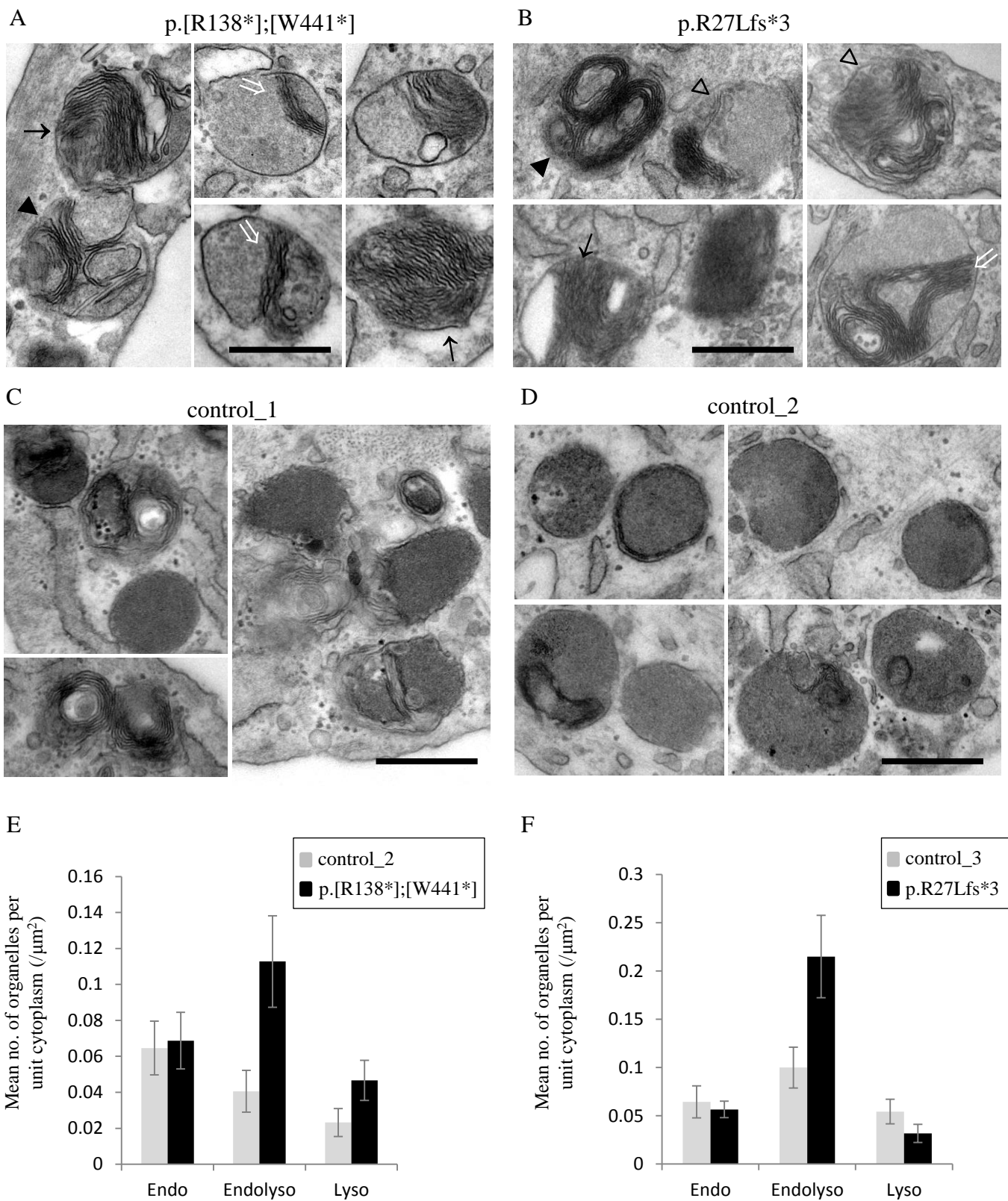

Figure S2

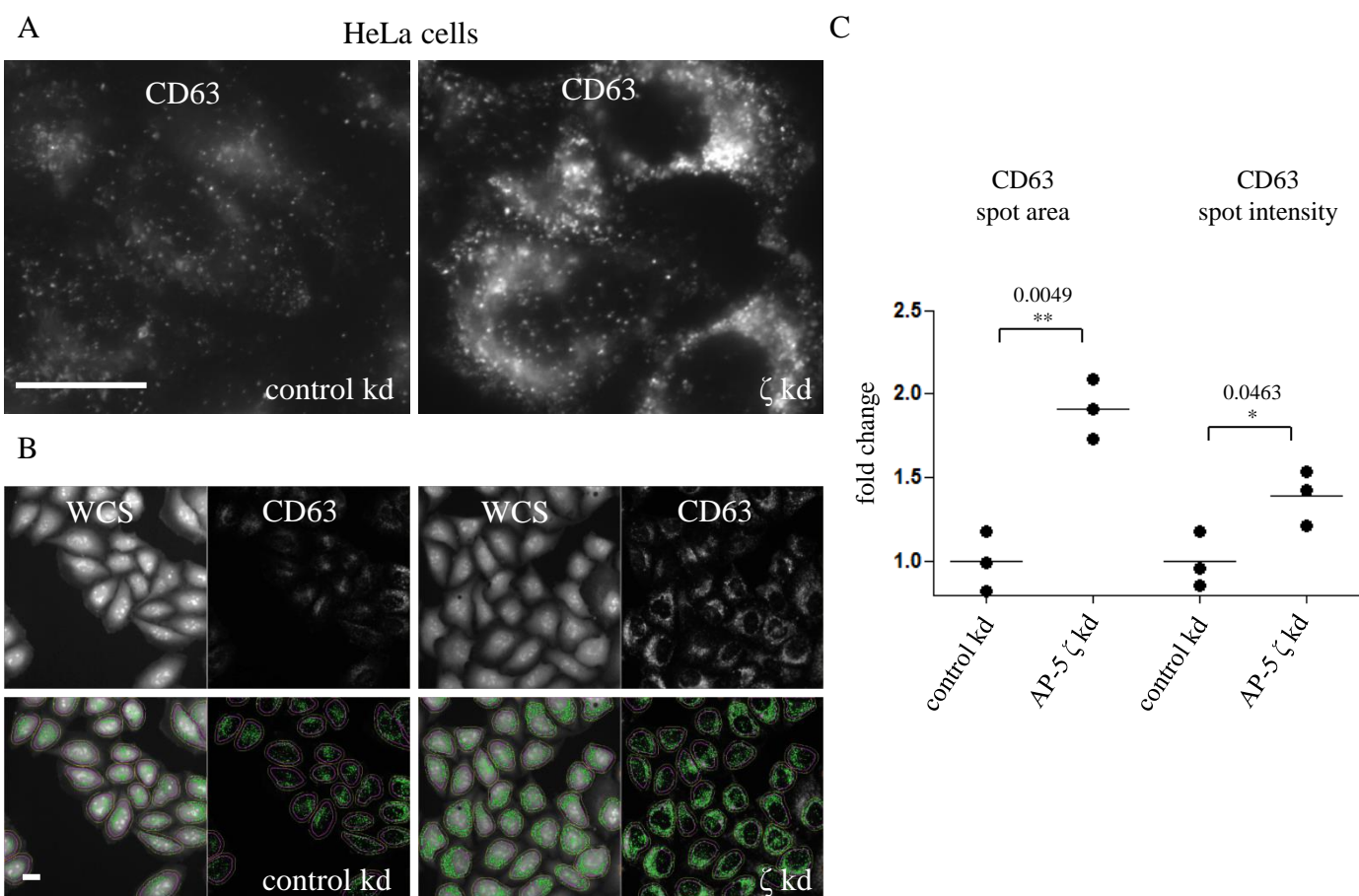

Figure S3

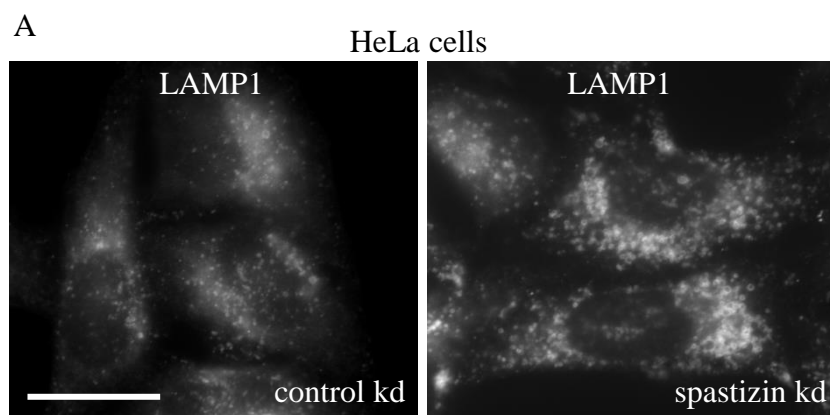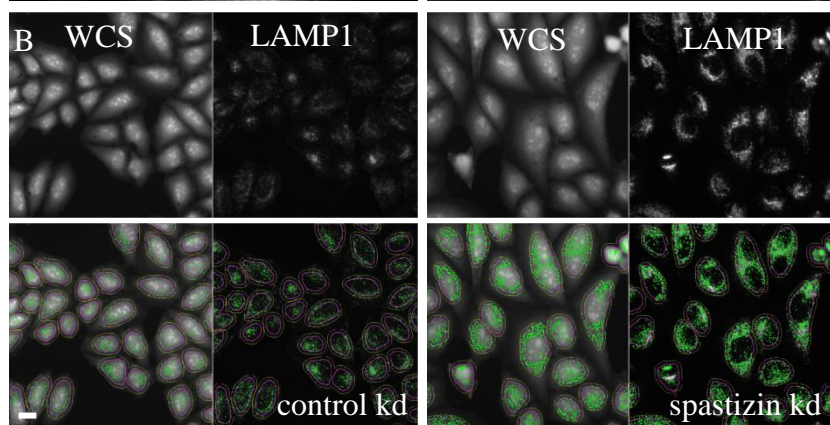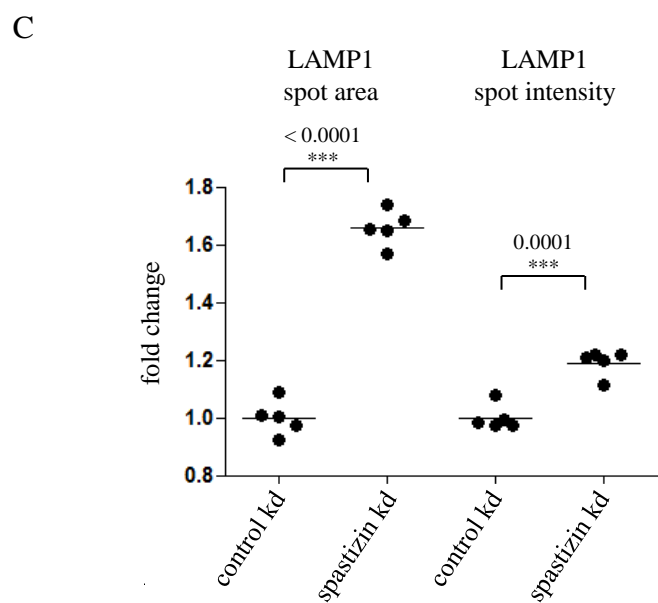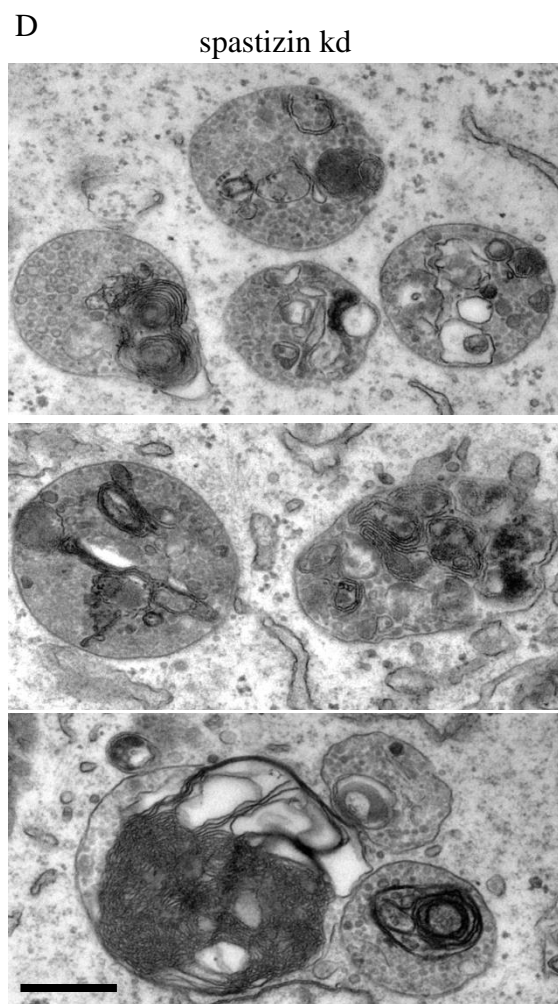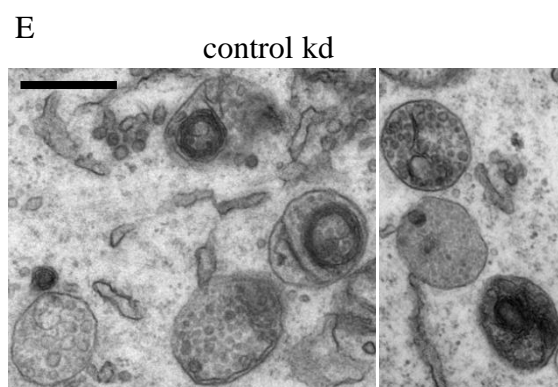

Figure S4

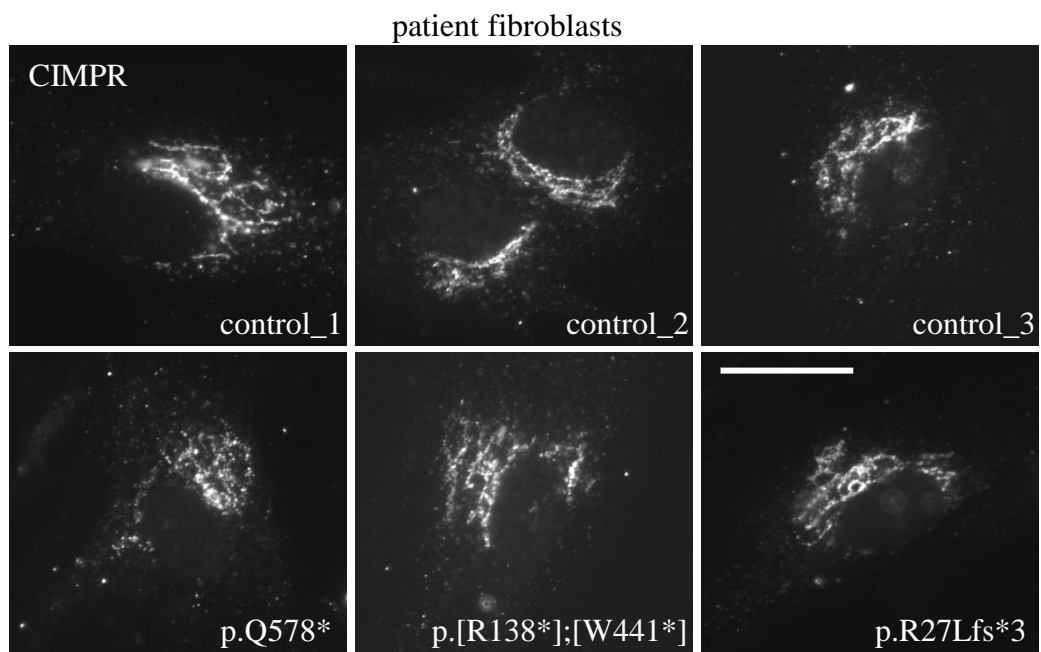

Figure S5

A HeLa cells

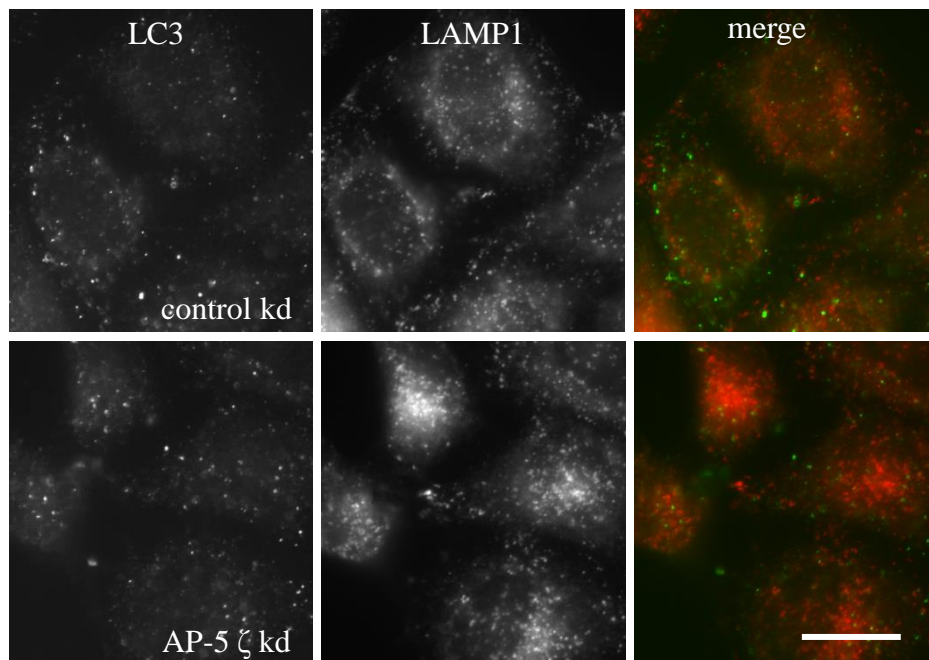

B patient fibroblasts

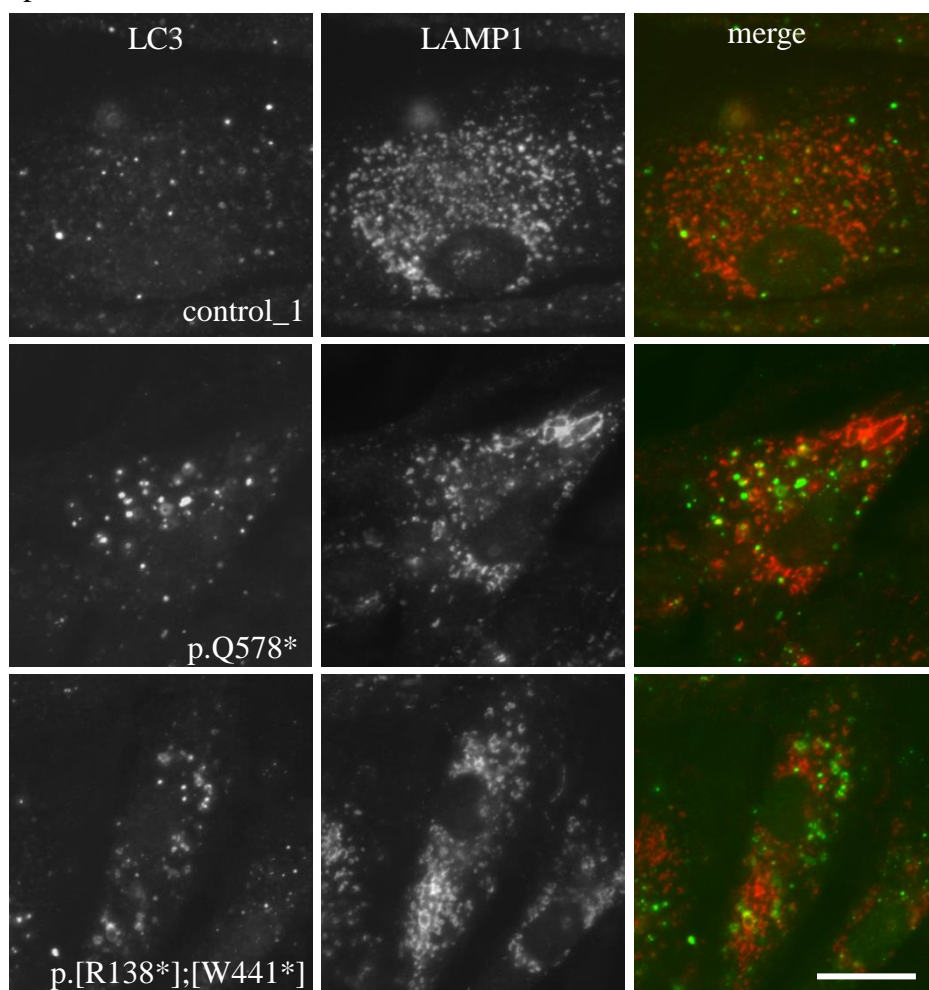

Figure S6

Supplement: Supplementary Data [file supp_ddv220_ddv220supp_figs.pdf]
