# Supplementary material for: Loss of AP-5 results in accumulation of aberrant endolysosomes: defining a new type of lysosomal storage disease
Source: Hum Mol Genet. 2015 Jun 17;24(17):4984–96. doi: 10.1093/hmg/ddv220 (PMC4527494; doi:10.1093/hmg/ddv220)
Supplement: Supplementary Data [file supp_ddv220_ddv220supp.doc]

**Supplemental Material Legends**

**Figure S1**. Expression patterns of AP-5. Two month-old mouse tissue lysates (**A**) or mouse brain tissue lysates at different ages (**B**) were loaded at equal protein level and then probed for AP-5 ζ and either actin or tubulin. Note that AP-5 is widely expressed, including different regions of the brain, and is expressed during different stages of development from embryonic to adulthood (E: Embryonic day; P: post-natal day). (**C**) Western blots of rat tissues lysates loaded at equal protein and probed for AP-5 ζ (arrow), and clathrin (CHC) as a reference point. Note that AP-5, like clathrin, is widely expressed and includes prominent expression in brain. (**D**) In situ hybridization of E15.5 embryos and adult 68 day old Wistar rats with a SPG48 (AP5Z1) antisense oligonucleotide showing expression of SPG48 in neurons in cortex, hippocampus (both dentate gyrus and ammon’s horn), cerebellum and pons. Three antisense probes were used as controls and showed no staining on cortex sections (one probe shown here). Scale bar = 50μm.

**Figure S2**. Ultrastructure of patient lines with mutations in *AP5Z1*. Electron microscopy was performed on patient-derived fibroblasts (denoted by their mutation); (**A**) p.[R138*];[W441*]; (**B**) p.R27Lfs*3; and control fibroblasts (**C**) gender- and age-matched with p.Q578*; (**D**) gender- and age-matched with p.[R138*];[W441*]. In all patient lines mutation of AP-5 ζ led to the accumulation of endocytic structures filled with aberrant storage material typified by multiple exaggerated membrane whorls (filled arrow head), belts of striated material (double arrow), fingerprint bodies (arrow), and some intraluminal vesicles (open arrow head). Note that these structures are predominantly surrounded by a single bilayer membrane. Scale bar = 500 nm. The number of different endocytic structures per unit area of cytoplasm was measured, where Endo = endosome (electron lucent lumen, may contain intraluminal vesicles, but no membrane whorls), Endolyso = endolysosome (contains membrane whorls, may contain intraluminal vesicles, or lucent or dark lumen), Lyso = lysosome (electron dark lumen, no membrane whorls or intraluminal vesicles). Images were systematically collected from 10 cells and the number of endocytic structures measured were 45 for control_2 and 72 for p.[R138*];[W441*] (**E**), and 73 for control_3 and 125 for p.R27Lfs*3 (**F**). Note the increase in the number of endolysosomes per unit area of cytoplasm in both patient lines.

**Figure S3**. AP-5 knockdown in HeLa cells. (**A**) HeLa cells were knocked down for AP-5 ζ or non-targeting control in a 2 hit 4 day protocol to achieve efficient knockdown, and then formaldehyde fixed and labelled with an antibody against CD63. Note that depletion of AP-5 ζ results in larger CD63 labelling. Scale bar = 20 μm. (**B**) HeLa cells knockdown for AP-5 ζ or non-targeting control were plated onto 96-well plates and labelled with a whole cell stain (WCS) and an antibody against CD63; the WCS allowed a mask to be drawn around the cells (pink) and various parameters of CD63 labelling (green) to be quantified on an ArrayScan Cellomics microscope using a SpotDetector algorithm. Scale bar = 20 μm. (**C**) Spot average intensity and average area per object were quantified in three independent experiments, with >1000 cells quantified per experiment. Note the significant increase in brightness and area of the CD63-positive puncta.

**Figure S4**. Spastizin knockdowns in HeLa cells. (**A**) HeLa cells were knocked down for spastizin or non-targeting control and then formaldehyde fixed and labelled with an antibody against LAMP1. Note that depletion of spastizin results in the increased brightness LAMP1 labelling, and the puncta appear larger. Scale bar = 20 μm. (**B**) and (**C**) HeLa cells knockdown for spastizin or non-targeting control were plated onto 96-well plates and labelled for whole cell stain (WCS) and LAMP1; the WCS allowed a mask (pink) to be drawn around the cells and various parameters of LAMP1 puncta (green) to be quantified on an ArrayScan Cellomics microscope using a SpotDetector algorithm. Scale bar = 20 μm. Spot average intensity and average area per object were quantified in three independent experiments, with >1000 cells quantified per experiment. Note the significant increase in brightness and area of LAMP1-positive puncta. Electron microscopy was performed on HeLa cells knockdown for spastizin (**D**) or non-targeting control (**E**). Note that the loss of spastizin results in the accumulation of enlarged morphologically defined endocytic structures filled with aberrant storage material. Scale bar = 500 nm.

**Figure S5**. Steady state localisation of CIMPR. Patient-derived fibroblasts cells were labelled with an antibody against CIMPR. Note that CIMPR labelling is very reticular and restricted to the perinuclear region, which is consistent with its localisation to the trans Golgi Network. Scale bar = 20 μm.

**Figure S6**. Enlarged endocytic structures are not LC3 positive. HeLa cells knocked down for AP-5 ζ (**A**), and patient-derived fibroblasts cells (**B**) were double labelled with antibodies against LAMP1 and LC3. Although most cells under these conditions were not undergoing autophagy, the enlarged LAMP1-positive structures are largely devoid of LC3 labelling. Scale bar = 20 μm.
